# M1 macrophage-derived CXCL12 drives neurogenic heterotopic ossification following spinal cord injury

**DOI:** 10.4103/NRR.NRR-D-25-01263

**Published:** 2026-02-05

**Authors:** Yulei Xie, Yaomin Luo, Xin Chen, Yinxu Wang, Wei Song, Hua Ling

**Affiliations:** 1 Department of Rehabilitation Medicine, Affiliated Hospital of North Sichuan Medical College, Nanchong, Sichuan Province, China; 2 School of Rehabilitation, Capital Medical University, Beijing, China; 3 Department of Rehabilitation Engineering, China Rehabilitation Science Institute, Beijing, China

**Keywords:** *CXCL12*, *CXCR4*, heterotopic ossification, macrophage, migration, mineralization, osteogenic, PI3K/AKT, proliferation, spinal cord injury

## Abstract

Patients with spinal cord injury frequently develop neurogenic heterotopic ossification, whose pathogenesis remains incompletely understood. Existing research models struggle to accurately simulate the complex pathological process. To establish a reliable neurogenic heterotopic ossification research model, elucidate its pathogenesis, and explore early intervention strategies, this study successfully developed a spinal cord injury-induced neurogenic heterotopic ossification mouse model. Significant ectopic bone formation, restricted hip and knee joint mobility, and motor dysfunction were observed, accompanied by elevated expression of the osteogenic markers alkaline phosphatase, runt-related transcription factor 2, sex-determining region Y-box 9, and osteocalcin. Proteomics and quantitative polymerase chain reaction analysis revealed upregulation of chemokine (C-X-C motif) ligand (*CXCL*)12, C-X-C chemokine receptor type 4 (*CXCR4*), and LYN proto-oncogene, whereas *CXCL1* was downregulated in the neurogenic heterotopic ossification group. *In vivo* experiments confirmed abnormal accumulation of M1 macrophages in muscles surrounding early ectopic bone tissue, with markedly elevated *CXCL12* expression. *In vitro* studies further revealed that M1 macrophages are the primary source of *CXCL12* secretion, and their cell culture supernatants promote the proliferation, migration, and osteoblastic differentiation potential of bone marrow mesenchymal stem cells. Mechanistically, *CXCL12* activates the phosphatidylinositol 3-kinase/protein kinase B pathway by binding to the *CXCR4* receptor, thereby driving the osteogenic differentiation of bone marrow mesenchymal stem cells. These findings indicate a causal relationship between the pathological process of neurogenic heterotopic ossification following spinal cord injury and the activation of the *CXCL12-CXCR4*-phosphatidylinositol 3-kinase-protein kinase B signaling axis, which modulates bone marrow mesenchymal stem cell function through M1 macrophage polarization. This study reveals a key mechanism driving the osteogenic differentiation of bone marrow mesenchymal stem cells, providing new directions for early warning and targeted treatment of neurogenic heterotopic ossification following spinal cord injury.

## Graphical abstract

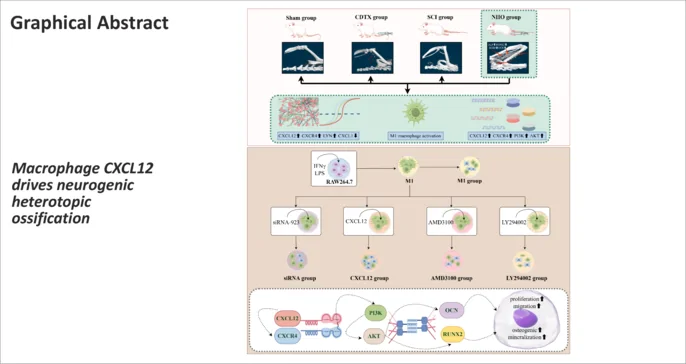

## Introduction

Currently, there are approximately 20 million patients with spinal cord injury (SCI) worldwide, with 10%–40% developing neurogenic heterotopic ossification (NHO) within 1–3 months post-injury (Ding et al., 2022). This complication significantly reduces functional independence scores (by 47.1%) by restricting joint mobility and encasing neurovascular bundles (Valbuena Valecillos et al., 2022), preventing clinically meaningful functional recovery in patients with SCI (Franz et al., 2022). Preventive measures, such as nonsteroidal anti-inflammatory drugs and bisphosphonates, have limited therapeutic windows and side effects (Tőkési et al., 2020), and over one-third of cases require surgical resection after 18–24 months of bone maturation, with recurrence rates as high as 19.8%–50% (Rizvi et al., 2022). Three key factors contribute to the challenges of NHO prevention and treatment: (1) absence of early biomarkers, as current imaging only detects mature NHO (Valbuena Valecillos et al., 2022); (2) complex pathogenesis involving neural–immune–bone interactions poorly replicated in animal models; and (3) unique mechanisms requiring consideration of muscle inflammation, macrophage activation, and neural signaling (Ampadiotaki et al., 2021). Therefore, establishing reliable *in vivo* models and elucidating the molecular drivers of NHO are imperative for developing early interventions.

Recent studies reveal SCI-induced neurogenic inflammation exhibits spatiotemporal specificity, with substance P/calcitonin gene-related peptide release within 72 hours triggering M1 macrophage infiltration and osteoblast differentiation (Tuzmen and Campbell, 2018). M1 macrophages further promote osteogenesis via interleukin-1β (IL)-1β)/IL-6 secretion (Genêt et al., 2015), whereas SCI severity correlates with NHO incidence, suggesting neural regulation of muscle microenvironment (Girard et al., 2021). However, the precise molecular mechanisms of this neural–immune–bone axis remain unclear.

**Highlights**

• A mouse model of heterotopic ossification following spinal cord injury was established, showing clinical-like phenotypes and motor dysfunction.

• M1 macrophages aggregate post-injury and secrete *CXCL12*, directly driving osteogenic differentiation of bone marrow stem cells.

• The *CXCL12-CXCR4-PI3K-AKT* pathway is a critical bridge linking macrophage activation to abnormal bone formation.

• The regulatory network from signaling molecules to cellular functions of heterotopic ossification following spinal cord injury was systematically deciphered.

• The *CXCL12-CXCR4-PI3K-AKT* pathway may serve as an early biomarker. Its inhibitors and macrophage modulators are promising novel therapies.

Bone marrowderived mesenchymal stem cells (BMSCs) are a major source of osteoprogenitors in heterotopic ossification, capable of migrating to injured soft tissues and differentiating into boneforming cells under proosteogenic cues (Chu et al., 2020; Chen et al., 2024). The chemokine (C–X–C motif) ligand (CXCL)12 and its receptor C–X–C chemokine receptor type 4 (*CXCR4*) play pivotal roles in stem cell homing, inflammatory recruitment, and bone regeneration. Notably, the phosphatidylinositol 3-kinase (*PI3K*)/protein kinase B (*AKT*) pathway acts as a downstream effector of *CXCL12/CXCR4* signaling and is involved in osteogenic differentiation of BMSCs (Thieme et al., 2009; Alexander et al., 2019). However, whether the *CXCL12-CXCR4-PI3K/AKT* axis participates in the pathogenesis of NHO following SCI, particularly in mediating the crosstalk between M1 macrophages and BMSCs, has not been investigated.

To bridge these knowledge gaps, we first established a reproducible mouse model of NHO by combining complete spinal cord transection with local muscle injury, which recapitulates the clinical progression of NHO. Using this model alongside proteomic screening, we identified *CXCL12* as a candidate regulator upregulated in early NHO lesions. We then hypothesized that M1 macrophagederived *CXCL12* promotes BMSC osteogenic differentiation via *CXCR4*-dependent activation of the *PI3K/AKT* pathway, thereby driving ectopic bone formation after SCI. Through integrated *in vivo* and *in vitro* approaches, this study aims to: (1) establish an inflammatory mouse model using micro-computed tomography (micro-CT) and proteomics to identify biomarkers; and (2) elucidate the *CXCL12-CXCR4-PI3K/AKT* signaling axis by which M1 macrophages promote BMSC osteogenic differentiation through combined *in vivo* and *in vitro* experiments. Our findings provide novel insights into the pathophysiology of NHO and highlight potential therapeutic targets for preventing this disabling complication.

## Methods

### Animal model establishment

Forty-eight specific pathogen-free C57BL/6 female mice (9–10 weeks old, 20–26 g) were purchased from Sibeifu Biotechnology Co., Ltd. (Beijing, China; license No. SCXK (Jing) 2019-0010). All procedures were approved by the Animal Experiments and Experimental Animal Welfare Committee of Capital Medical University (approval No. AEEI-2024-168, June 21, 2024) and complied with the ARRIVE 2.0 guidelines (Percie du Sert et al., 2020).

After 1 week of acclimatization, the mice were randomly divided into Sham, cardiotoxin (CDTX), SCI, and NHO groups (*n* = 12 per group). A modified Hassan Shaker spinal cord transection method was used to establish a complete SCI model (Shaker et al., 2003). The Sham group underwent laminectomy only. CDTX, a myotoxin rich in phospholipase A2 extracted from cobra venom, triggers a typical inflammatory cascade response, which is critical for heterotopic ossification (HO) induction (Garry et al., 2016). CDTX (125 µg/mL in phosphate-buffered saline, 0.1 mL; Alomone Labs, Jerusalem, Israel, Cat# C-205) or phosphate-buffered saline (0.1 mL, 1×, Gibco; Cambridge, MA, USA, Cat# 10010023) was bilaterally injected into hamstring muscles.

### Behavioral assessment

Basso Mouse Scale (BMS) scores (Fang et al., 2021) were assessed on days 1, 7, 14, and 28 post-surgery by two blinded researchers following standard protocols (0 = complete paralysis, 9 = normal locomotion). Higher scores indicated better limb function.

### Maximum passive joint range of motion

The range of motion (ROM) at the knee and hip joints of the right hind limb was measured at 1, 7, 14, and 28 days post-modeling for each group. According to the skeletal structure of the mouse hindlimb, four custom-made labels of different colors were affixed to the iliac crest, hip joint, knee joint, and ankle joint of anesthetized mice. The mice were placed in a prone position on a mouse board, with a 0.5 N weight suspended at the ankle joint of the right hindlimb (Minamimoto et al., 2021). A camera positioned 20 cm from the right hind limb captured images, and ImageJ software (version 1.52/Java 18.0_112; National Institutes of Health, Bethesda, MD, USA) (Schneider et al., 2012) measured hip and knee joint ROMs in the captured images.

### Micro-CT analysis

After the mice were anesthetized with isoflurane (3%–4% for induction, 1%–2% for maintenance; Sigma-Aldrich, St. Louis, MO, USA, Cat# I4049), micro-CT (μCT 40; SCANCO Medical, Brüttisellen, Switzerland) was performed at 70 kV/114 μA with 300 ms integration time. The scanning resolution was 16 μm. CTAn software (version 1.17.7.2; SCANCO Medical) was used to measure the NHO volume of each mouse, with gray-scale threshold levels set at 45 and 220 Hounsfield units. These thresholds were determined by phantom calibration and histomorphometric validation (Debaud et al., 2017). The ectopic lamellar bone formation volume was quantitatively assessed using mouse in situ skeletal subtraction technology to detect and quantify NHO (Alexander et al., 2022).

### Tissue collection

At 7, 14, and 28 days after modeling, four mice were randomly selected from each group, euthanized under excessive anesthesia (3%–4% for induction, 1%–2% for maintenance; Sigma-Aldrich), and dissected. The hind limbs were separated at the femoral neck, and the paws were removed at the ankle joint. After measurement and weighing, the tissues were rapidly frozen in liquid nitrogen (LINDE, Munich, Germany) and stored at –80°C.

### Cell culture

The RAW264.7 cell line is widely used in macrophage polarization research. RAW264.7 cells (Keycell Biotechnology Co., Ltd., Wuhan, China, Cat#QS-M003, RRID: CVCL_0493) were cultured in Dulbecco’s modified Eagle medium (Gibco, Cat# 11995065) supplemented with 10% fetal bovine serum (Servicebio, Wuhan, China, Cat# G8003) at 37°C and 5% CO_2_. M1 macrophage polarization was induced by treatment with interferon-γ (100 ng/mL; PeproTech, Cranbury, NJ, USA, Cat# 315-05) and lipopolysaccharide (Sigma, Cat# L2880, 100 ng/mL) (Munteanu et al., 2020).

### Flow cytometry

Flow cytometric analysis was performed to characterize M1 phenotype RAW264.7 macrophages. Cells were stained with fluorescently labeled antibodies against the macrophage marker F4/80 (BioLegend, San Diego, CA, USA, Cat# 123116) and M1-specific surface marker CD86 (BioLegend, Cat# 105008). Following standard staining protocols, samples were analyzed using a flow cytometer (Accuri C6; BD Biosciences). Data acquisition and analysis were performed with Accuri CFlow Plus software (version 1.0.227.4; BD Biosciences).

### Cell transfection

M1 macrophages were transfected with small interfering RNA (siRNA) and siRNA-negative control (NC) targeting CXCL12 (RiboBio, Guangzhou, China, Cat# siB1706200429) (**Table 1**), PI3K inhibitor (LY294002; Selleck, Houston, TX, USA, Cat# S1105, 10 μM), or CXCR4 inhibitor (AMD3100; MedChemExpress, Monmouth Junction, NJ, USA, Cat# HY-10046, 10 μM). siRNA and siRNA-NC were transfected using Lipofectamine 2000 (Invitrogen, Cat# 11668019).

**Table 1:** Small interfering RNA targeting *CXCL12* mRNA

Gene	Forward sequence (5'–3')	Reverse sequence (5'–3')
*siRNA-370*	GGA GAA AGC UUU AAA CAA GTT	CUU GUU UAA AGC UUU CUC CTT
*siRNA-923*	GGG AGG CUC CUU UAU CCA GTT	CUG GAU AAA GGA GCC UCC CTT
*siRNA-NC*	UUC UCC GAA CGU GUC ACG UTT	ACG UGA CAC GUU CGG AGA ATT

*siRNA-370*: Small interfering RNA-370; *siRNA-923*: small interfering RNA-923; *siRNA-NC*: small interfering RNA-negative control.

### Osteogenic induction

Mouse BMSCs (Zishan Biotech, Wuhan, China, Cat#STCC6011P) were cultured in osteoblastic differentiation induction medium (Procell, Wuhan, China, Cat# CM-M121) with 5% CO_2_ at 37°C to induce osteogenesis. On the fifth or sixth day of induction, osteoblastic differentiation induction medium, M1 macrophage supernatant, or treated M1 macrophage supernatant was added. Samples were collected on the seventh day of osteogenic induction for detection.

### Histological staining

Fresh hamstring muscle tissue was immediately immersed in 4% paraformaldehyde fixative (Solarbio, Beijing, China, Cat# G1120) for 24 hours. The tissue was trimmed using a sterile scalpel (Feather, Osaka, Japan, Cat# 10-688), followed by dehydration through a graded ethanol series (70%, 95%, and 100%; Sigma-Aldrich), paraffin embedding (Sigma-Aldrich, Cat# P3803), paraffin sectioning (5 μm thickness), and slide sealing (Thermo, Cat# 12-565-81).

Paraffin sections were dewaxed through xylene (Sigma-Aldrich, Cat# 27025) and rehydrated to distilled water. Staining was performed using Mayer’s hematoxylin (Beyotime, Shanghai, China, Cat# C0105) for 5 minutes, followed by eosin Y (Beyotime, Cat# C0107) counterstaining after blue staining with 1% ammonia water. Slides were dehydrated through an ethanol series, cleared in xylene, and mounted with neutral balsam (BKMAM, Changsha, China, Cat# B1201).

Paraffin-embedded sections were processed through sequential steps: antigen retrieval using citrate buffer (Beyotime, Cat# P0083, pH 6.0, 95°C, 20 minutes), endogenous peroxidase blocking with 3% H_2_O_2_ (Beyotime, Cat# P0203, room temperature, 10 minutes), nonspecific binding blocking by 5% bovine serum albumin (Solarbio, Cat# A8020, room temperature, 30 minutes), primary antibody incubation [anti-alkaline phosphatase (ALP), mouse, 1:200, Abcam, Cambridge, UK, Cat# ab108337; anti-runt-related transcription factor 2 (RUNX2), rabbit, 1:200, Abcam, Cat# ab76956; anti-sex-determining region Y-box 9 (SOX9), rabbit, 1:200, Abcam, Cat# ab185966; anti-osteocalcin (OCN), rabbit, 1:200, Abcam, Cat# ab93876; all 4°C overnight], horse radish peroxidase-conjugated secondary antibody (Beyotime, Cat# A0208, 1:500, 37°C, 1 hour); 3,3′-diaminobenzidine chromogenic development (Beyotime, Cat# P0202, 3–5 minutes), hematoxylin counterstaining (1 minute), and mounting with neutral balsam (BKMAM, Cat# B1201). Finally, the images were observed and analyzed using a fluorescence microscope (BX53; Olympus, Tokyo, Japan). The integrated optical density was quantitatively analyzed using ImageJ software.

### Quantitative polymerase chain reaction

Total RNA was extracted from the harvested hamstring muscle tissue or the treated BMSCs using TRIzol reagent (Invitrogen, Carlsbad, CA, USA, Cat# 15596026). The quality and concentration of RNA samples were determined by measuring absorbance at 260 nm/280 nm using a NanoDrop^TM^ 2000 spectrophotometer (Thermo Fisher Scientific, Waltham, MA, USA). Reverse transcription was performed using GoScript^TM^ reverse transcriptase (Promega, Madison, MI, USA, Cat# A5000). Quantitative polymerase chain reaction (qPCR) experiments were conducted using PowerUp^TM^ SYBR^TM^ Green Master Mix (Thermo, Cat# A25742) according to the manufacturer’s protocols. Primer sequences are shown in **Table 2**. Gene expression levels were calculated using the comparative cycle threshold method (Livak and Schmittgen, 2001).

**Table 2:** Gene primer sequence

Gene	Forward sequence (5'–3')	Reverse sequence (5'–3')
*GAPDH*	CCT CGT CCC GTA GAC AAA ATG	TGA GGT CAA TGA AGG GGT CGT
*ALP*	GGC ACC TGC CTT ACC AAC TCT	GTT GTG GTG TAG CTG GCC CTT A
*RUNX2*	ATG ACA CTG CCA CCT CTG ACT TCT	AGG GAT GAA ATG CTT GGG AAC T
*SOX9*	GCA GAC CAG TAC CCG CAT CT	TCC GCT TGT CCG TTC TTC AC
*OCN*	GGA GGG CAA TAA GGT AGT GAA CAG	ATAG CTC GTC ACA AGC AGG GT
*AKT1*	TCT GCC CTG GAC TAC TTG C	CTT CTC GTG GTC CTG GTT G
*PI3K*	GAC CAA TAC TTG ATG TGG CTG ACG	CTC GCA ATA GGT TCT CCG CTT T
*CXCL12*	CGC CCA GAC AGA AGT CAT AGC	CCT TGC CTT TGT TCA GTA TCT TTT G
*CXCR4*	GCT AAG GAG CAT GAC GGA CAA	CTG ACT GTT GGT GGC GTG GA
*LYN*	AGT GCA GGA GCT TTC CTT ATC A	CGA GGA GAG ATG TAA TAG CCA CC
*MAPK3*	TCC GCC ATG AGA ATG TTA TAG GC	GGT GGT GTT GAT AAG CAG ATT GG
*CXCL1*	GCA TCT GGT GAA GGT GGT G	GGA GGT GGA GGT GGA GGT
*CYBB*	CCT CCT ATG ACT TGG AAA TG GA	CCT TCT GTT GAG ATC GCC A

*ALP*: Alkaline phosphatase; *AKT1*: AKT serine/threonine kinase 1; *CXCL1*: C–X–C motif chemokine ligand 1; *CXCL12*: C–X–C motif chemokine ligand 12; *CXCR4*: C–X–C motif chemokine receptor 4; *CYBB*: cytochrome b-245 beta chain; *GAPDH*: glyceraldehyde-3-phosphate dehydrogenase; *LYN*: LYN proto-oncogene, Src family tyrosine kinase; *MAPK3*: mitogen-activated protein kinase 3; *OCN*: osteocalcin; *RUNX2*: Runt-related transcription factor 2; *PI3K*: phosphatidylinositol 3-kinase; *SOX9*: SRY-box transcription factor 9.

### Mass spectrometry

Total proteins were extracted from the harvested hamstring muscle tissue using radioimmunoprecipitation assay buffer (Beyotime, Cat# P0013B) supplemented with protease and phosphatase inhibitors (Roche, Basel, Switzerland, Cat# 04693132001). Protein concentrations were quantified using a bicinchoninic acid assay kit (Thermo, Cat# 23225), and protein quality was verified by sodium dodecyl sulfate–polyacrylamide gel electrophoresis (Bio-Rad, Hercules, CA, USA, Cat# 4561096). Following protein reduction (Sigma, Cat# 43815)/alkylation (Sigma, Cat# I1149), tryptic digestion (Promega, Cat# V5111), peptide desalting (Thermo, Cat# 89852), and quantification, liquid chromatography–tandem mass spectrometry analysis was performed using an EASY-nLC 1200 system (Thermo, Cat# 164534) coupled with a timsTOF Pro2 mass spectrometer (Bruker, Billerica, MA, USA) to generate DIA data-independent acquisition raw data (Luo, 2023). RAW files were processed using Spectronaut software (version 19.6; Biognosys, Zurich, Switzerland) (Niu et al., 2022), and database searches were conducted against the UniProt mouse database (release 2023_11) using Spectronaut software (version 19.6). The search settings were as follows: enzyme specificity was set to trypsin (full), allowing for up to two missed cleavages; fixed modification was carbamidomethylation of cysteine; and variable modifications included methionine oxidation, and N-terminal acetylation. The precursor mass tolerance was set to 10 ppm and the fragment ion mass tolerance to 0.02 Da. The false discovery rate for both peptide and protein identification was set to < 1%.

### Bioinformatics analysis and quantitative polymerase chain reaction validation

Differentially expressed proteins were identified using the *t*-test function in R (R Core Team, Vienna, Austria) (|log2fold change| > 1, *P* < 0.05). Protein–protein interaction networks were constructed via STRING (Szklarczyk et al., 2023) and visualized with Cytoscape (version 3.10; Cytoscape Consortium). Core genes were identified using CytoHubba (version 0.1) (Wang et al., 2025). Relative expression levels of the hub genes in the transcriptomic sequencing dataset (GSE94683, GEO database) were retrieved. Receiver operating characteristic analysis was performed according to NHO status, and hub genes with area under the curve values > 0.7 were selected for qPCR validation in animal models. Primer sequences (Sangon Biotech, Shanghai, China, high-performance liquid chromatography-purified) are shown in **Table 1**.

### Immunofluorescence staining

Paraffin sections were dewaxed to water, or the treated BMSCs were prepared. After antigen retrieval (citrate buffer, Beyotime, Cat# P0083), samples were incubated with primary antibodies [anti-cluster of differentiation (CD)68, mouse, 1:200, Shanghai Fusheng Industrial Co., Ltd., Shanghai, China, Cat# AK16947; anti-inducible nitric oxide synthase, rabbit, 1:100, Thermo, Cat# 18943-1-AP, RRID: AB_2879936; anti-CD206, rabbit, 1:500, Thermo, Cat# 60143-1-Ig, RRID: AB_2879936; anti-CXCL12, rabbit, 1:1000, Thermo, Cat# 17402-1-AP, RRID: AB_2879936) at 4°C overnight, followed by fluorescently labeled secondary antibodies at room temperature for 1 hour]. Nuclei were stained with 4′,6-diamidino-2-phenylindole (Beyotime, Cat# C1005), and anti-fluorescence decay mounting agent (Beyotime, Cat# P0128) was applied. Samples were observed under a fluorescence microscope, and images were acquired. Average fluorescence intensity was calculated using ImageJ.

### Western blot assay

Total proteins were extracted from the treated BMSCs using radioimmunoprecipitation assay buffer (Beyotime, Cat# P0013B) supplemented with protease and phosphatase inhibitors (Roche, Cat# 04693132001). Primary antibodies against CXCL12 (1:200; Bioss, Beijing, China, Cat# bs-20347R, AB_2884144), CXCR4 (1:200; Bioss, Cat# bs-1013R, RRID: AB_2884145), PI3K (1:200; Bioss, Cat# bs-1068R, RRID: AB_2884146), AKT1 (1:200; Bioss, Cat# bs-0115R, RRID: AB_2884147), RUNX2 (1:200; Bioss, Cat# bs-1134R, RRID: AB_2884148), and OCN (1:200; Bioss, Cat# bs-4917R, RRID: AB_2884149) were incubated overnight at 4°C. Glyceraldehyde-3-phosphate dehydrogenase (GAPDH; Xianzhi Biotech, Hangzhou, China, Cat# AB-P-R 001, RRID: AB_2884144) was used as a loading control for normalization (Zhou et al., 2023). The corresponding secondary antibody (anti-mouse immunoglobulin G, 1:200; BD Biosciences, Franklin Lakes, NJ, USA, Cat# 770937, RRID: AB_11157747) was incubated for 1 hour at room temperature. Western blotting results were demonstrated by showing representative blots alongside quantitative bar graphs. Band signals were detected by chemiluminescence, captured digitally, and analyzed by measuring the grayscale intensity using ImageJ. The relative expression of each target protein was calculated after normalization to glyceraldehyde-3-phosphate dehydrogenase.

### Cell Counting Kit-8 assay

After BMSC culture, 10 μL of Cell Counting Kit-8 reagent (Beyotime, Cat# CK04) was added to each well and incubated at 37°C for 2 hours. Optical density at 450 nm was measured using a microplate reader (Synergy H1; BioTek, Winooski, VT, USA). Cell viability was calculated according to the manufacturer’s instructions using the formula: Cell survival rate (%) = (experimental well absorbance – blank well absorbance)/(control well absorbance – blank well absorbance) × 100.

### Cell migration

BMSCs were seeded and cultured using Transwell chambers (Corning, Corning, NY, USA, Cat# 3422). Cells were fixed with 70% ice-cold ethanol solution (Sigma, Cat# 459836) and stained with 0.5% crystal violet solution (Sigma, Cat# C0775). Non-migrated cells on the upper chamber side were removed before microscopic analysis (IX73; Olympus). Cell migration was manually quantified using ImageJ.

### Alkaline phosphatase staining

ALP activity was assessed by visualizing blue–black ALP in cells as follows: BMSCs smears were fixed and treated with the ALP staining working solution (Beyotime, Cat# C3206); the working solution was removed, and samples were dehydrated using anhydrous ethanol (Sigma, Cat# 459844), cleared with xylene (Sigma, Cat# 534056), and sealed with neutral resin (Solarbio, Cat# G8590); and the slides were examined under an inverted microscope for staining intensity comparison and distribution pattern analysis.

### Alizarin red staining

Calcium deposition was evaluated by Alizarin red staining. BMSCs were fixed, incubated with Alizarin red staining working solution (Solarbio, Cat# G1450), washed, and examined under an inverted microscope for staining intensity comparison and distribution pattern analysis.

### Enzyme-linked immunosorbent assay

Enzyme-linked immunosorbent assay was performed according to the manufacturer’s instructions (R&D Systems, Minneapolis, MN, USA, Cat# DY200). After sequential centrifugation (5424R; Eppendorf, Hamburg, Germany) to clarify the supernatant from BMSCs cultured with or without M1 macrophages supernatant, dilution, plate coating, washing, addition of biotinylated antibody (1:200) and enzyme conjugate working solutions, incubation, washing, tumor mutation burden substrate (Sigma, Cat# T0440) reaction, and termination with sulfuric acid (Sigma, Cat# 258105), absorbance at 450 nm was measured by an enzyme-labeled instrument.

### Statistical analysis

Data were analyzed using SPSS (version 26.0; IBM Corp., Armonk, NY, USA) and GraphPad Prism (version 10.1.2; GraphPad Software, Boston, MA, USA) software. Quantitative data are presented as mean ± standard deviation or standard error. Comparisons among multiple groups were performed using one-way analysis of variance with *post hoc* Tukey’s test, comparisons between two groups were performed using an independent samples *t*-test, and comparisons within groups were performed using a paired *t*-test. *P* < 0.05 was considered statistically significant.

## Results

### Decreased motor ability in neurogenic heterotopic ossification mice

On postoperative days 1, 7, 14, and 28, the BMS scores for the Sham and CDTX groups were 9 points at all time points, whereas those for the SCI and NHO groups were 0 points, indicating successful establishment of a complete SCI mouse model (**Table 3**).

**Table 3:** Basso Mouse Scale scores of mice with neurogenic heterotopic ossification following spinal cord injury

Group	Day 1 (*n* = 48)	Day 7 (*n* = 48)	Day 14 (*n* = 32)	Day 28 (*n* = 16)
Sham	9±0	9±0	9±0	9±0
CDTX	9±0	9±0	9±0	9±0
SCI	0	0	0	0±0
NHO	0	0	0	0±0

Data are expressed as mean ± standard deviation. CDTX: Cardiotoxin; NHO: neurogenic heterotopic ossification; SCI: spinal cord injury.

On postoperative day 1, no significant differences were observed in the maximum ROMs of the hip (*P* = 0.879) and knee (*P* = 0.74) between groups, indicating that the different modeling methods had minimal initial impact on joint mobility. During the subsequent observation period, the maximum hip and knee ROMs in the Sham and CDTX groups remained unchanged. In the SCI group, compared with postoperative day 1, no significant reduction was observed in maximum knee ROM on postoperative day 7, whereas the maximum hip ROM showed a significant decrease (*P* < 0.001). This decrease persisted and further worsened on postoperative days 14 and 28 (*P* < 0.001), indicating that SCI progressively impaired hip and knee joint mobility. In the NHO group, the maximum hip and knee ROMs were significantly reduced on postoperative day 7 (*P* < 0.001) and further decreased on postoperative days 14 *(P* < 0.001) and 28 (*P* < 0.001). By postoperative day 28, the maximum hip and knee joint ROMs in the NHO group had decreased to 95.21° ± 6.5° and 92.71° ± 1.76°, respectively, which were significantly lower than those in the SCI group (106.89° ± 7.64° and 98.71° ± 3.63°, respectively; *P* < 0.05), suggesting that NHO further exacerbates the SCI-induced reduction in joint mobility (**Tables 4** and **5**).

**Table 4:** Range of motion at the knee joints of mice with neurogenic heterotopic ossification following spinal cord injury

Group	Day 1 (*n* = 48)	Day 7 (*n* = 48)	Day 14 (*n* = 32)	Day 28 (*n* = 16)
Sham	117.51±4.72	120.11±5.01	118.46±5.95	116.21±6.77
CDTX	118.63±6.80	120.32±3.83	121.07±4.73	117.59±4.33
SCI	115.46±7.80	115.12±4.74^#^	105.56±5.18^###^	98.71±3.63^###^
NHO	117.78±5.88	101.95±3.09^###^	99.76±2.84^###^	92.71±1.76^###^

*F*	0.527	49.804	36	0.836
*P*-value	0.666	< 0.001	< 0.001	< 0.001

Data are expressed as mean ± standard deviation. ^#^*P* < 0.05, ^###^*P* < 0.001, *vs*. Sham group (one-way analysis of variance followed by *post hoc* Tukey’s test). CDTX: Cardiotoxin; NHO: neurogenic heterotopic ossification; SCI: spinal cord injury.

**Table 5:** Range of motion at the hip joints of mice with neurogenic heterotopic ossification following spinal cord injury

Group	Day 1 (*n* = 48)	Day 7 (*n* = 48)	Day 14 (*n* = 32)	Day 28 (*n* = 16)
Sham	130.39±2.37	130.93±2.74	131.27±2.68	131.35±1.86
CDTX	129.40±3.01	129.91±2.59	130.76±2.55	131.04±4.97
SCI	130.52±2.68	116.93±4.56^###^	114.81±8.29^###^	106.89±7.64^###^
NHO	129.51±3.24	114.71±7.41^###^	103.43±3.21^###^	95.21±6.56^###^

*F*	0.505	38.451	62.67	40.242
*P*-value	0.681	< 0.001	< 0.001	< 0.001

Data are expressed as mean ± standard deviation. ^###^*P* < 0.001, *vs*. Sham group (one-way analysis of variance followed by *post hoc* Tukey’s test). CDTX: Cardiotoxin; NHO: neurogenic heterotopic ossification; SCI: spinal cord injury.

### Morphological and structural changes in the popliteal muscle tissue and formation of ectopic lamellar bone

**Figure 1:**
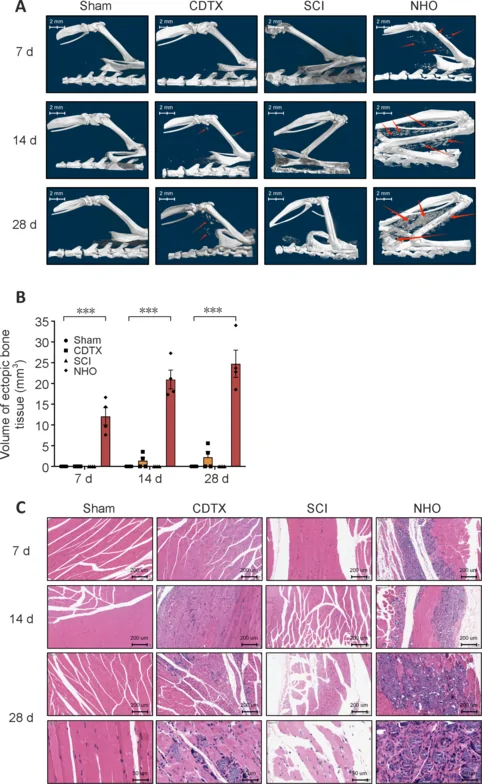
Morphological and structural changes in the popliteal muscle tissue and formation of heterotopic lamellar bone in the NHO mice. (A) Three-dimensional bone tissue images obtained by micro-CT. In the NHO group, ectopic ossification progressed markedly over time (7 days: scattered calcification foci; 14 days: foci fusion; 28 days: mature lamellar bone throughout the region). The CDTX group developed only minimal ossification at 7 days and 14 days. No ectopic mineralization was observed in the other two groups at any time point. with red arrows indicating ectopic bone tissues. Scale bars: 2 mm in A. (B) Quantitative analysis of bone tissue over time. (C) Hematoxylin and eosin staining of hamstring muscles tissue. In the NHO group, extensive inflammatory infiltration, fibroblastic proliferation, and immature woven bone with osteoid seams were observed at postoperative day 7. By day 28, the tissue had matured into extensive, interconnected plates of lamellar bone. None of the other three groups exhibited similar pathological changes at any time point. Scale bars: 200 μm and 50 μm in C. Data are expressed as mean ± SEM (*n* = 4/group). ***P < 0.001 (one-way analysis of variance followed by *post hoc* Tukey’s test). CDTX: Cardiotoxin; NHO: neurogenic heterotopic ossification; SCI: spinal cord injury.

Micro-CT analysis revealed that in the NHO group, all mice exhibited numerous scattered calcification foci in the popliteal muscle region of the hind limbs on postoperative day 7. By postoperative day 14, calcification foci had begun to expand and fuse. By postoperative day 28, ectopic lamellar bone formation had spread throughout the entire hamstring region of the hind limbs (**Figure 1A**). The volume of ectopic bone tissue increased from 12.86 ± 4.69 mm^3^ and 21.91 ± 5.02 mm^3^ to 25.09 ± 7.98 mm^3^ (postoperative days 7, 14, and 28, respectively; **Figure 1B**). Hematoxylin and eosin staining demonstrated that, in the NHO group, hamstring tissue exhibited extensive inflammatory infiltration accompanied by collagen-like connective tissue formation on postoperative day 7. Fibroblast proliferation and hypertrophy, capillary proliferation, and immature woven bone with osteoid seams containing regions with osteoblasts and chondrocytes were observed. Extensive trabecular bone and partial medullary cavity structures were also present, with regions rich in various leukocytes within the medullary cavity, and some cavities containing proliferating undifferentiated mesenchymal cells. By postoperative day 28, extensive maturation of trabecular bone and connection into plates was observed (**Figure 1C**).

### Upregulation of osteogenic marker expression in neurogenic heterotopic ossification mice

**Figure 2:**
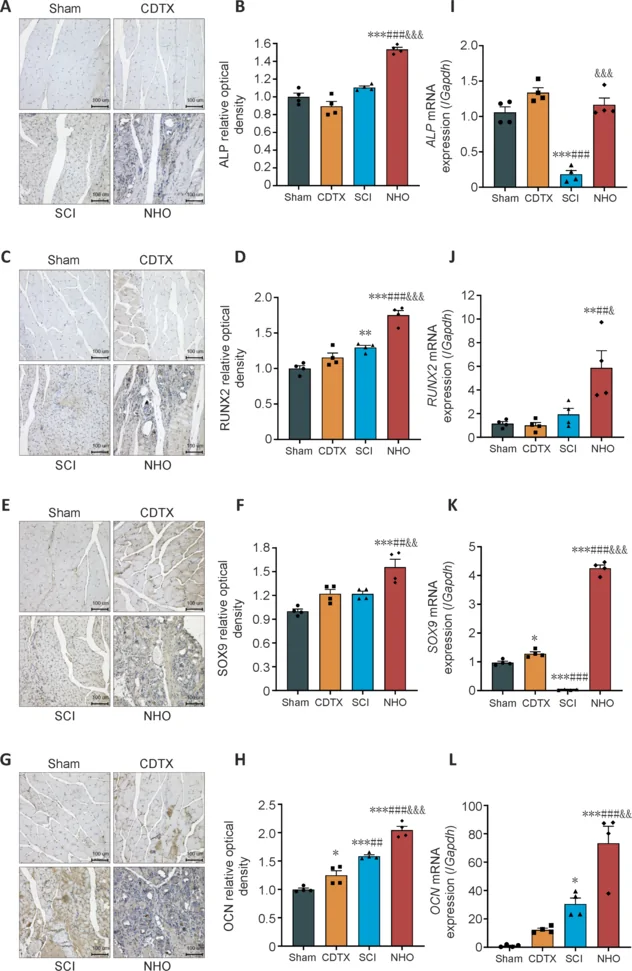
Osteogenic markers are highly expressed in the hamstring muscle tissue of NHO mice. (A, C, E, G) Immunohistochemical staining images of ALP, RUNX2, SOX9, and OCN in the hamstring muscle tissue. On postoperative day 7, their expression was mildly elevated in the CDTX and SCI groups, and markedly increased in the NHO group, compared with the sham group. Scale bars: 100 μm in A, C, E, G. (B, D, F, H). The relative expression levels of ALP, RUNX2, SOX9, and OCN in the hamstring muscle tissue compared to the Sham group. (I–L) mRNA expression of *ALP, RUNX2*, *SOX9*, and *OCN* in the hamstring muscle tissue. Data are expressed as mean ± SEM (*n* = 4/group). *P < 0.05, **P < 0.01, ***P < 0.001, *vs*. Sham group; ##*P* < 0.01, ###*P* < 0.001, *vs*. CDTX group; &*P* < 0.05, &&*P* < 0.01, &&&*P* < 0.001, *vs*. SCI group (one-way analysis of variance followed by *post hoc* Tukey’s test). ALP: Alkaline phosphatase; CDTX: cardiotoxin; GAPDH: glyceraldehyde-3-phosphate dehydrogenase; NHO: neurogenic heterotopic ossification; OCN: osteocalcin; RUNX2: Runt-related transcription factor 2; SOX9: sex-determining region Y-box 9; SCI: spinal cord injury.

Immunohistochemical staining of hamstring muscle tissues revealed that, compared with the sham group, the expression of osteogenic markers (ALP, SOX9, RUNX2 and OCN) increased in the CDTX and SCI groups on postoperative day 7, whereas expression in the NHO group increased significantly. The relative fluorescence intensities of osteogenic markers in the NHO group were significantly higher than those in the Sham group (**Figure 2A–H**). Moreover, ALP and SOX9 mRNA expression in the NHO group was higher than in the SCI group (*P* < 0.05; **Figure 2I** and **K**), and RUNX2 and OCN mRNA expression in the NHO group was higher than in the Sham (*P* < 0.05) and CDTX groups (*P* < 0.01; **Figure 2J** and **L**). Overall, osteogenic marker expression in the NHO group was higher than in the SCI group.

### Identification and validation of hub genes involved in neurogenic heterotopic ossification following spinal cord injury

**Figure 3:**
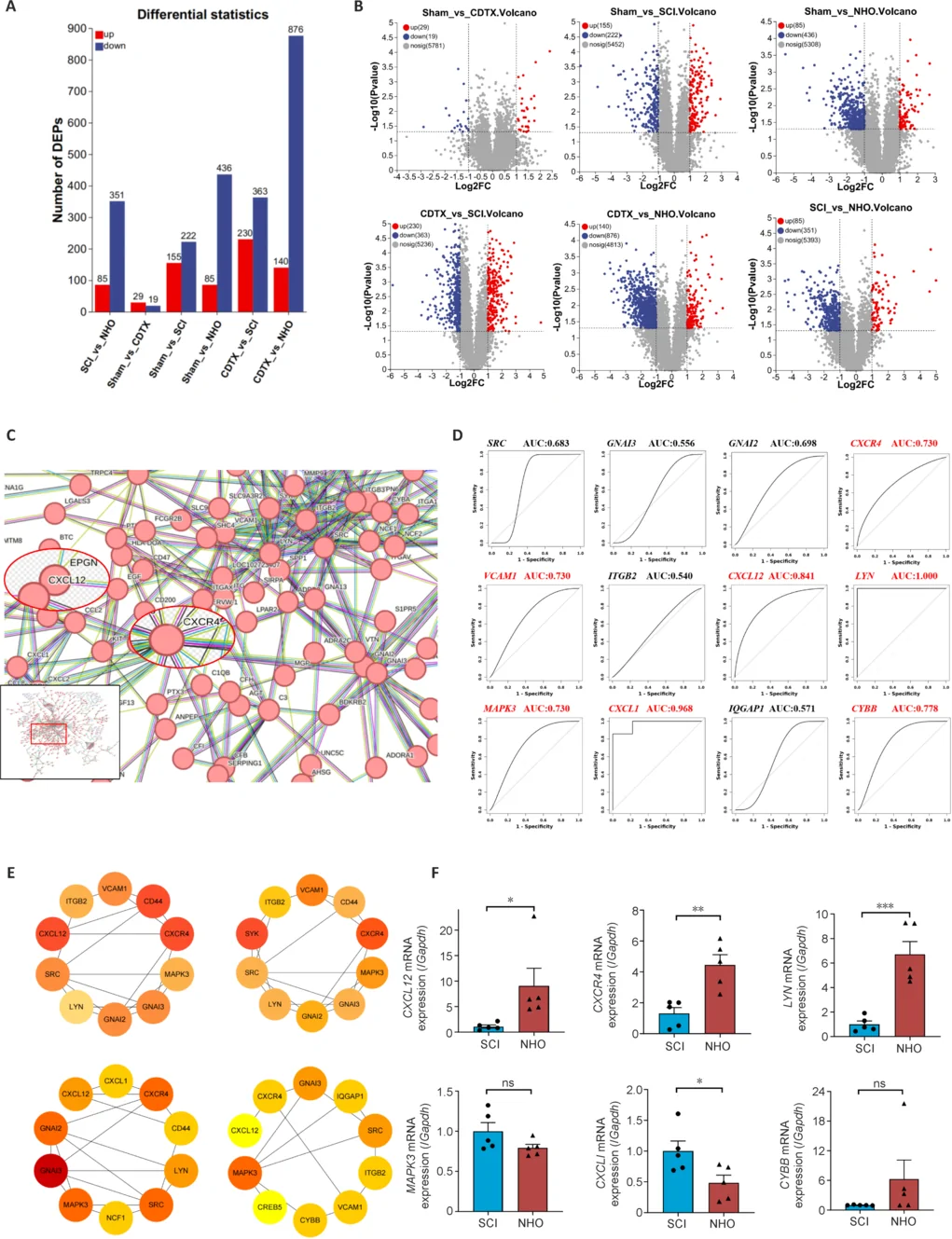
Identification and validation of hub genes involved in neurogenic heterotopic ossification following spinal cord injury. (A) Number of DEPs. (B) Volcano plot of DEPs. (C) PPI network construction. (D) Interaction diagram of hub genes screened by four different algorithms. (E) ROC analysis of hub genes, with genes with AUC > 0.7 marked in red. (F) PCR results of hub genes. Data are expressed as mean ± SEM (*n* = 5/group). *P < 0.05, **P < 0.01, ***P < 0.001, *vs.* SCI group (unpaired two-tailed *t*-test). AUC: Area under curve value; CDTX: cardiotoxin; CXCL1: C–X–C motif chemokine ligand 1; CXCL12: C–X–C motif chemokine ligand 12; CXCR4: C–X–C motif chemokine receptor 4; CYBB: cytochrome b-245 beta chain; DEP: differentially expressed protein; GAPDH: glyceraldehyde-3-phosphate dehydrogenase; LYN: LYN proto-oncogene, Src family tyrosine kinase; MAPK3: mitogen-activated protein kinase 3; NHO: neurogenic heterotopic ossification; ROC: receiver operating characteristic; SCI: spinal cord injury.

Differentially expressed protein screening revealed that, compared with the SCI group, the NHO group had 351 significantly upregulated proteins and 85 significantly downregulated proteins. Compared with the Sham group, the NHO group had 436 significantly upregulated proteins and 85 downregulated proteins. Compared with the CDTX group, the NHO group had 876 significantly upregulated proteins and 140 downregulated proteins (**Figure 3A** and **B**). When the differentially expressed proteins were input into STRING to construct a protein–protein interaction network, *CXCL12* and *CXCR4* were found to occupy relatively central positions (**Figure 3C**). Protein interaction data were imported into Cytoscape, and the top 10 hub genes were identified using four algorithms (maximal clique centrality, maximum neighborhood component, density of maximum neighborhood component, and degree centrality) (**Figure 3D**). The four algorithms yielded 12 hub genes in total. Receiver operating characteristic curve analysis revealed that the area under curve for seven hub genes [*CXCR4*, vascular cell adhesion molecule 1, *CXCL12*, LYN proto-oncogene (*LYN*), mitogen-activated protein kinase 3, *CXCL1*, and *CYBB*] was greater than 0.7, indicating a significant correlation with NHO occurrence after SCI (**Figure 3E**). Further qPCR analysis revealed that on postoperative day 14, compared with the SCI group, the NHO group exhibited significantly increased expression levels of *CXCL12* (*P* < 0.05), *CXCR4* (*P* < 0.01), and *LYN* (*P* < 0.01) in hamstring muscle tissue, whereas *CXCL1* expression was significantly downregulated (*P* < 0.05). There were no significant differences observed in mitogen-activated protein kinase 3 and *CYBB* expression levels (*P* > 0.05; **Figure 3F**). These findings suggest that *CXCL12*, *CXCR4*, *LYN*, and *CXCL1* are hub genes significantly associated with NHO occurrence following SCI.

### Macrophage infiltration and M1 polarization accompanied by high expression of *CXCL12*, *CXCR4*, *PI3K*, and *AKT* in muscle surrounding ossified tissue

**Figure 4:**
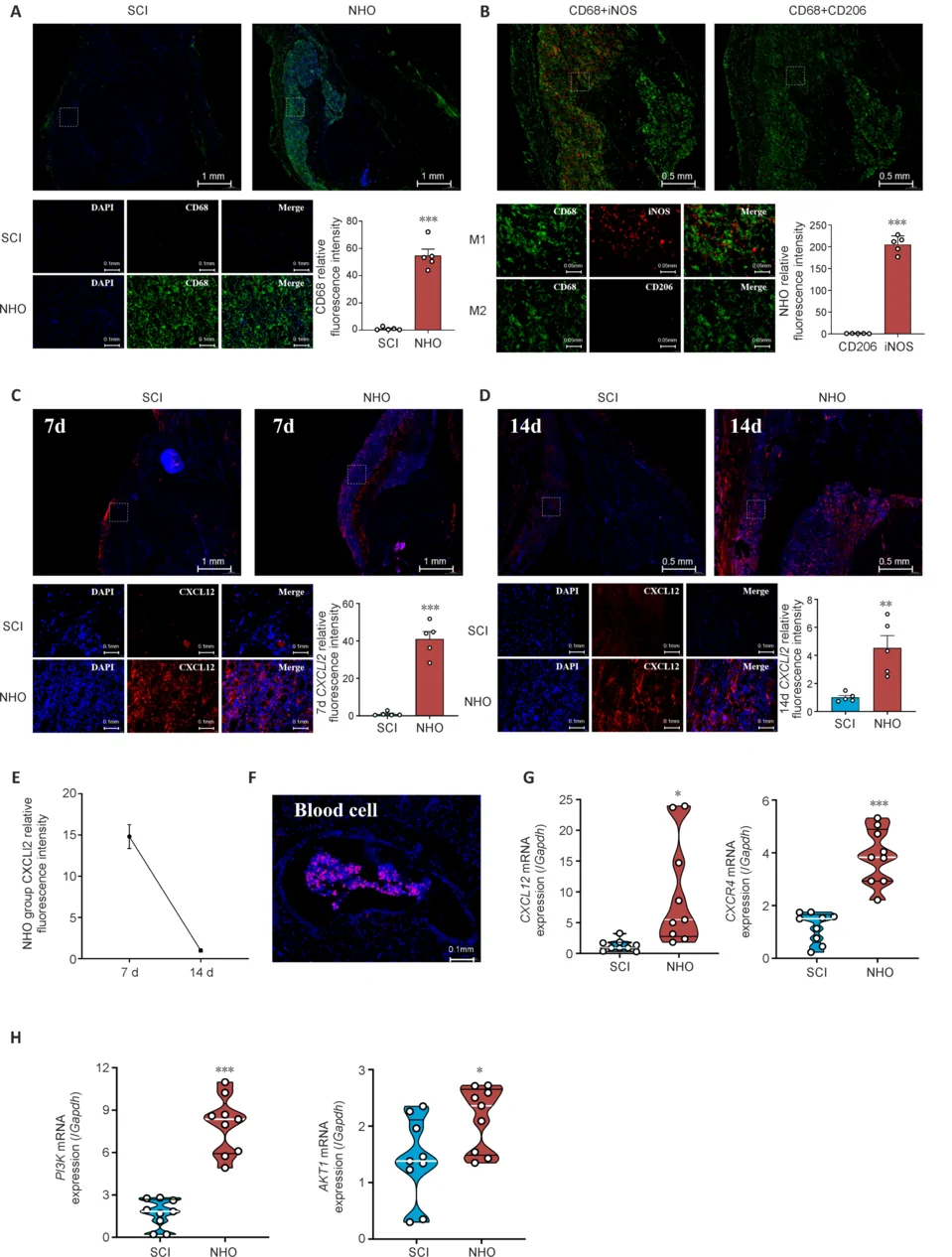
Macrophage infiltration and M1 polarization accompanied by high expression of the *CXCL12*, *CXCR4*, *PI3K*, and *AKT* in the muscle surrounding ossified tissue. (A) CD68 (green) immunofluorescence representative the NHO group showed significantly higher CD68^+^ cell density and fluorescence intensity in hamstring muscle than the SCI group on postoperative day 7. Scale bars: 1 mm and 0.1 mm. (B) Co-localization immunofluorescence of CD68 (green)/iNOS (red) (M1 marker) and CD68 (green)/CD206 (red) (M2 marker) in NHO group muscles at 7 days. Scale bars: 0.5 mm and 0.05 mm. (C, D) *CXCL12* expression was significantly elevated in the NHO group versus the SCI group at both day 7 and day 14. Scale bars: 1 mm and 0.1 mm. (E) Time-course analysis of CXCL12 fluorescence intensity in NHO group. (F) CXCL12 (red) immunofluorescence in vascular blood cells. Scale Scale bars: 1 mm and 0.1 mm. (G) The relative expression of *CXCL12/CXCR4* at 7 days (*n* = 9/group). (H) Violin plots for *PI3K/AKT1* expression at 7 days (*n* = 9/group). Data are expressed as mean ± SEM. *P < 0.05, **P < 0.01, ***P < 0.001, *vs.* CD206 group or SCI group (unpaired two-tailed *t*-test). AKT1: AKT serine/threonine kinase 1; CD206: cluster of differentiation 206; CD68: cluster of differentiation 68; CXCL12: C-X-C motif chemokine ligand 12; GAPDH: glyceraldehyde-3-phosphate dehydrogenase; iNOS: inducible nitric oxide synthase; NHO: neurogenic heterotopic ossification; PI3K: phosphatidylinositol 3-kinase; SCI: spinal cord injury.

Immunofluorescence analysis revealed that, on postoperative day 7, CD68-positive cell counts and fluorescence intensity in hamstring muscle tissue of the NHO group were significantly higher than in the SCI group (*P* < 0.001; **Figure 4A**), indicating substantial macrophage infiltration at the site of HO. Further analysis revealed that inducible nitric oxide synthase (an M1 marker; Mei et al., 2024) fluorescence intensity was significantly higher than that of CD206 (an M2 marker; Ono et al., 2021) (*P* < 0.001; **Figure 4B**), indicating that pro-inflammatory M1 macrophages predominated during the early stage of NHO formation. Concurrently, *CXCL12* fluorescence intensity in the NHO group was significantly higher than in the SCI group (*P* < 0.01; **Figure 4C**) and remained elevated on postoperative day 14 (*P* < 0.01; **Figure 4D**), although it decreased significantly from its peak on postoperative day 7 (*P* < 0.01; **Figure 4E**), suggesting a critical role for *CXCL12* in the early stages of NHO formation. Notably, *CXCL12* expression increased within blood vessels on postoperative day 14 (**Figure 4F**), indicating potential systemic ossification signaling via the circulatory system. qPCR confirmed significantly higher *CXCL12* and *CXCR4* mRNA expression in ossified tissues of the NHO group compared with the SCI group (*P* < 0.01; **Figure 4G**). Furthermore, mRNA expression of key molecules in the *PI3K*/*AKT* pathway (*PI3K*, and *AKT1*) was significantly upregulated (*P* < 0.05; **Figure 4H**). These findings collectively support the involvement of the *CXCL12-CXCR4-PI3K-AKT* signaling axis in the pathological process of NHO formation following SCI.

### Effects of M1-type macrophage-derived *CXCL12* on osteogenic differentiation of bone marrow mesenchymal stem cells

**Figure 5:**
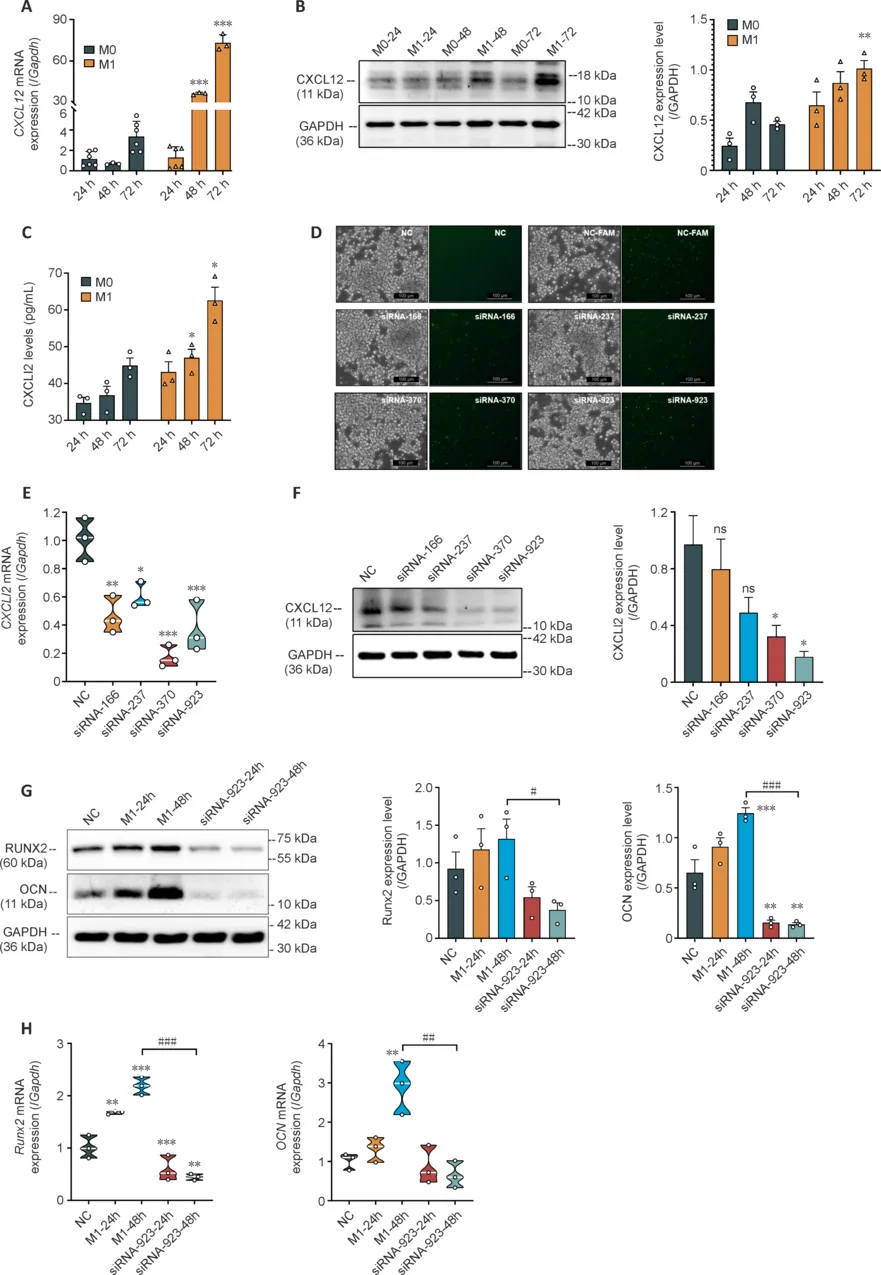
Effect of CXCL12 derived from M1-type macrophages on the osteogenic differentiation of BMSCs. (A) Relative expression levels of CXCL12 in M0- and M1-induced macrophage. (B) Western blot results and quantitative analysis of relative expression levels of CXCL12 in M0- and M1 macrophage groups. (C) The levels of CXCL12 in the supernatant at different time points in the M0 and M1 groups. (D) Immunofluorescence staining results of CXCL12 (green) in M1-type macrophages after transfection with different siRNAs. Scale bars: 100 μm. (E) Violin plot of the relative expression levels of *CXCL12* in M1-type macrophages after transfection with different siRNAs. (F) Western blot results of CXCL12 and quantitative analysis of relative expression levels of CXCL12 in M1-type macrophages after transfection with different siRNAs. (G) Western blot results and quantitative analysis of relative expression levels of RUNX2 and OCN proteins. (H) qPCR results of RUNX2 and OCN expression levels. Data are expressed as mean ± standard error (*n* = 3). *P < 0.05, **P < 0.01, ***P < 0.001, *vs.* M0 groups (A–C) or control group (E–H); #*P* < 0.05, ##*P* < 0.01, ###*P* < 0.001, *vs.* M1-48h groups (one-way analysis of variance followed by *post hoc* Tukey’s test)). BMSCs: bone marrow mesenchymal stem cells; CXCL12: C–X–C motif chemokine ligand 12; GAPDH: glyceraldehyde-3-phosphate dehydrogenase; NC: normal control; RUNX2: Runt-related transcription factor 2; OCN: osteocalcin; qPCR: quantitative polymerase chain reaction; siRNA: small interfering RNA.

Flow cytometry revealed that after M1 polarization induction, the proportion of CD86-positive cells in the M1 group was significantly higher than in the M0 group (*P* < 0.001; **Additional Figure 1**), indicating successful M1 polarization of macrophages. Western blotting, qPCR, and immunofluorescence analyses collectively demonstrated that the expression level of *CXCL12* in M1 macrophages was significantly higher than in M0 macrophages at 72 hours (*P* < 0.01; **Figure 5A** and **B**), indicating that M1 polarization induces *CXCL12* upregulation. The *CXCL12* content in the supernatant of the M1 group at 48 and 72 hours (M1-24h and M1-48h, respectively) was significantly higher than in the M0 group (*P* < 0.05, **Figure 5C**), indicating increased secretion of *CXCL12* by M1-polarized macrophages. Moreover, mRNA expression levels of *CXCL12* in M1-type macrophages transfected with siRNA-370 and siRNA-923 were significantly reduced (*P* < 0.05; **Figure 5D** and **E**). *CXCL12* protein expression was lowest in cells transfected with siRNA-923 (**Figure 5F**). These results demonstrate that siRNA-923 exhibited the strongest inhibitory effect on *CXCL12* and therefore was selected for subsequent experiments. Osteogenic marker analysis showed that compared with the NC group, mRNA and protein expression of RUNX2 and OCN was significantly increased in the M1-24h and M1-48h groups (*P* < 0.01). After transfection, the RUNX2 and OCN mRNA and protein expression levels were reduced in the siRNA-923-48h group. At 48 hours, significant differences were observed in the mRNA and protein expression of RUNX2 and OCN mRNA between the M1-48h and siRNA-923-48h groups (*P* < 0.05, **Figure 5G** and **H**), suggesting that *CXCL12* upregulates the expression of early osteogenic (RUNX2) and late osteogenic (OCN) markers in BMSCs.

### Effects of M1-type macrophage-derived *CXCL12* on downstream gene expression in osteogenic differentiation of bone marrow mesenchymal stem cells

**Figure 6:**
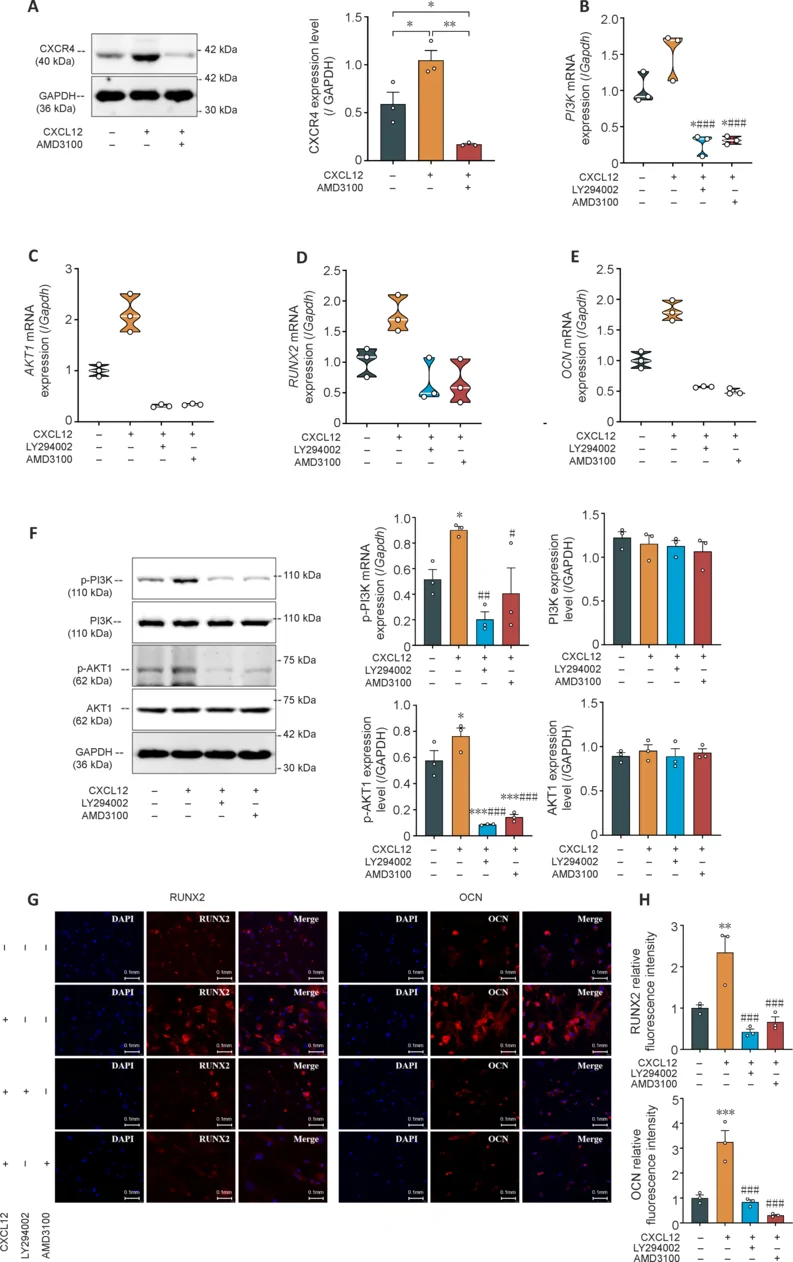
Effects of M1-type macrophage-derived CXCL12 on downstream gene expression. (A) Western blot results and relative expression levels of CXCR4 protein in BMSCs under different treatment conditions. (B, C) qPCR results of *PI3K* and *AKT1* in BMSCs under different treatment conditions. (D, E) qPCR detection results of *RUNX2* and *OCN* in BMSCs under different treatment conditions. (F) Western blot results and quantitative analysis of expression levels of phosphorylated PI3K and phosphorylated AKT1 in BMSCs under different treatment conditions. (G, H) Immunofluorescence staining images and quantitative analysis results of RUNX2 (red) and OCN (red) in BMSCs under different treatment conditions. Scale bars: 0.1 mm in G. Data are expressed as mean ± standard error (*n* = 3). *P < 0.05, **P < 0.01, ***P < 0.001, *vs.* control group; #*P* < 0.05, ##*P* < 0.01, ###*P* < 0.001, *vs.* CXCL12-treated group (one-way analysis of variance followed by *post hoc* Tukey’s test). AMD3100: A CXCR4 inhibitor; AKT1: AKT serine/threonine kinase 1; CXCR4: C-X-C motif chemokine receptor 4; GAPDH: glyceraldehyde-3-phosphate dehydrogenase; LY294002: a PI3K inhibitor; PI3K: phosphatidylinositol 3-kinase; qPCR: quantitative polymerase chain reaction; RUNX2: Runt-related transcription factor 2; OCN: osteocalcin.

Western blotting revealed that compared with the control group, CXCR4 expression was significantly upregulated in the *CXCL12*-treated group (*P* < 0.05). After treatment with the *CXCR4* receptor antagonist AMD3100, CXCR4 expression significantly decreased, approaching control levels (*P* < 0.001; **Figure 6A**), suggesting that *CXCL12* significantly upregulates *CXCR4* expression in BMSCs, whereas AMD3100 effectively antagonizes this effect. Combined western blotting, qPCR, and immunofluorescence analyses demonstrated that compared with the control group, the mRNA expression levels of *PI3K*, *AKT1*, *RUNX2*, and *OCN*, and the protein expression levels of p-PI3K and p-AKT1, were significantly upregulated in the *CXCL12*-treated group (*P* < 0.05). These levels were significantly reduced after AMD3100 treatment, suggesting that *CXCL12* regulates the *PI3K-AKT1* pathway and osteogenic factor expression in BMSCs via *CXCR4*. Additionally, treatment with the PI3K pathway inhibitor LY294002 significantly decreased the expression of *PI3K*, *AKT1*, *RUNX2*, and *OCN* (*P* < 0.001), suggesting that *CXCL12* promotes osteogenic factor expression in BMSCs through the *PI3K/AKT1* signaling pathway (**Figure 6B–H**). These results indicate that *CXCL12* activates the *PI3K/AKT* pathway via the *CXCR4* receptor, thereby significantly promoting the expression of *RUNX2* and *OCN* in BMSCs.

### M1-type macrophages promote the proliferation, migration, and osteogenic differentiation of bone marrow mesenchymal stem cells through the *CXCL12-CXCR4-PI3K-AKT* signaling axis

**Figure 7:**
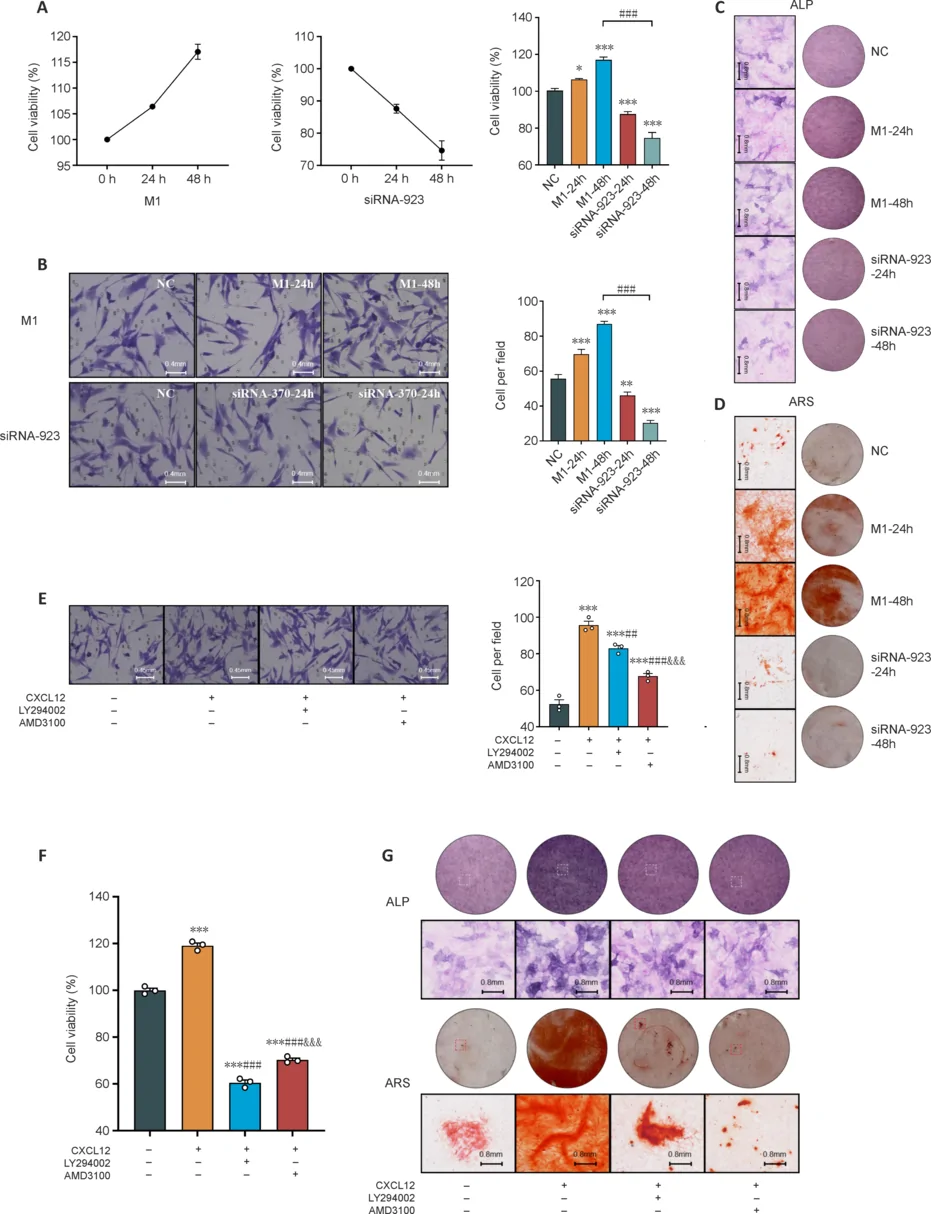
M1-type macrophages promote the proliferation, migration, and osteogenic differentiation of BMSCs through the *CXCL12-CXCR4-PI3K-AKT* signaling axis. (A) Changes in cell viability and statistical results after co-culturing BMSCs with M1-type macrophages or the supernatant of M1-type macrophages transfected with siRNA-370 for 0, 24, and 48 hours. (B) Microscopic images (magnification: 200×) from the Transwell migration assay and a bar chart showing the quantitative analysis of cell migration numbers. Scale bars: 0.4 mm. (C, D) ALP and ARS staining images and microscopic images of BMSCs co-cultured with the supernatant from M1 macrophages and M1 macrophages transfected with siRNA-923. M1 macrophage-conditioned medium upregulated ALP/ARS staining in BMSCs, an effect reversed by siRNA-923. Scale bars: 0.8 mm. (E) Transwell migration assay images and quantitative analysis of cell migration numbers under different treatment conditions. CXCL12 promoted BMSC migration via the CXCR4/PI3K axis, as these effects were blocked by AMD3100 or LY294002. Scale bars: 0.4 mm. (F) Cell viability of BMSCs under different treatment conditions. Data are expressed as mean ± standard error (*n* = 3). *P < 0.05, **P < 0.01, ***P < 0.001, *vs*. control group; ##*P* < 0.01, ###*P* < 0.001, *vs.* CXCL12-treated group; &&&*P* < 0.001, *vs.* LY294002 group (one-way analysis of variance). (G) ALP and ARS staining results of BMSCs under different treatment conditions. CXCL12 promoted BMSC osteogenesis and mineralization via the CXCR4/PI3K axis, as these effects were blocked by AMD3100 or LY294002. Scale bars: 0.8 mm. AKT: AKT serine/threonine kinase; ALP: alkaline phosphatase staining; AMD3100: a CXCR4 inhibitor; ARS: Alizarin Red S staining; BMSCs: bone marrow mesenchymal stem cells; CXCL12: C–X–C motif chemokine ligand 12; CXCR4: C–X–C motif chemokine receptor 4; LY294002: a PI3K inhibitor; NC: normal control; PI3K: phosphatidylinositol 3-kinase; siRNA: small interfering RNA.

Cell Counting Kit-8 assay and Transwell migration results demonstrated that compared with the NC group, the M1-24h and M1-48h groups exhibited significantly increased cell proliferation activity and migration (*P* < 0.01), which gradually increased over time, whereas the siRNA923-24h and siRNA-923-48h groups exhibited significantly reduced cell proliferation activity and migration (*P* < 0.01), with the decrease becoming more pronounced over time (**Figure 7A** and **B**), indicating that inhibiting the expression of *CXCL12* in M1-type macrophages significantly reduced the proliferation and migration of BMSCs. Compared with the NC group, the ALP and Alizarin red staining intensities were significantly increased in the M1-24h and M1-48h groups. After transfection with siRNA-923, *CXCL12* secretion in the supernatant decreased, and ALP and Alizarin red staining intensities were significantly reduced (**Figure 7C** and **D**), indicating that *CXCL12* promotes the osteogenic activity and mineralization potential of BMSCs. Compared with the control group, the *CXCL12*-treated group showed significantly increased cell viability, migration capacity, osteogenic activity, and mineralization capacity (*P* < 0.05). These effects were substantially attenuated by the CXCR4 receptor antagonist AMD3100 and the PI3K pathway inhibitor LY294002 (*P* < 0.05; **Figure 7E–G**). These results indicate that *CXCL12* activates the *PI3K/AKT* pathway through the CXCR4 receptor, significantly promoting the proliferation, migration, and osteogenic differentiation of BMSCs.

## Discussion

This study successfully established a stable mouse model of NHO following SCI by combining complete spinal cord transection with locally induced muscle injury using CDTX. The reliability and stability of this model were validated through comprehensive assessments of behavioral, motor function, imaging, and histological parameters, providing an important experimental platform for in-depth investigation of the pathophysiological mechanisms underlying NHO following SCI.

The formation of NHO involves three key elements: oligodendrocyte precursor cells, the local tissue microenvironment, and neural system regulation (Alexander et al., 2020). In this study, the expression of osteoblastic differentiation-related proteins was significantly higher in the NHO group than in the Sham, CDTX, and SCI groups, consistent with findings reported by Xiong (2022) regarding high expression of osteoblastic differentiation proteins in traumatic HO. These results suggest that NHO may share certain molecular pathways with traumatic HO, although its regulatory hierarchy appears to be more complex. Numerous studies have shown that a damaged central nervous system can release osteogenic induction signals via the circulatory system to the injury site (Cadosch et al., 2010), thereby promoting NHO formation. In the present study, only a small proportion of mice in the CDTX group developed ectopic lamellar bone formation, with the volume of ectopic ossification representing only 0%–10% of that observed in the NHO group. These findings indicate that the inflammatory microenvironment resulting from localized muscle injury is necessary for NHO formation, whereas SCI significantly elevates systemic neuroinflammatory levels, thereby significantly promoting NHO development at the site of muscle injury.

Macrophages are key immune cells closely involved in inflammatory responses and tissue repair. In this study, animal experiments confirmed significant macrophage infiltration in damaged muscle tissue during the early stages of NHO, with M1-type macrophages playing a central role in NHO development. M1-type macrophages promote osteogenic gene expression by secreting pro-inflammatory factors, such as IL-1β and tumor necrosis factor-α, which activate the bone morphogenetic protein and hypoxia-inducible factor-1α pathways (Tuzmen et al., 2018). Additionally, vascular endothelial growth factor released by M1-type macrophages not only promotes angiogenesis, providing a metabolic foundation for NHO, but also forms a pathological vascular network that facilitates oligodendrocyte precursor cell migration (Yang et al., 2012). During the later stages of NHO development, M1 macrophages coordinate bone repair and vascular maturation through anti-inflammatory factors, such as transforming growth factor (TGF)-β1 and IL-10, and the TGF-β1- mothers against decapentaplegic homolog 2/3 signaling pathway (Wang et al., 2018). Moreover, neurogenic factors and M1 macrophage polarization exhibit synergistic effects. Neither SCI nor muscle injury alone is sufficient to induce NHO, but when combined, they can mediate NHO, suggesting that the persistent neuroinflammation induced by SCI can alter the local microenvironment, promoting M1 polarization and excessive activation of macrophages, ultimately leading to amplified osteogenic signaling cascades (Shi and Pamer, 2011).

*CXCL12* exhibits time-dependent dynamic changes during NHO development following SCI, with high expression in muscle tissue surrounding early heterotopic bone, peaking at approximately 40 times that of normal muscle tissue. Its expression gradually decreases as bone matures, reaching four or five times that of normal muscle tissue by day 14, and normalizes by the mature stage of ectopic lamellar bone, suggesting that *CXCL12* primarily acts during the initiation of NHO. Additionally, this is the first study to observe abnormally elevated *CXCL12* expression levels in blood cells within the vasculature of SCI-induced NHO model mice, suggesting that *CXCL12* may spread systemically via the bloodstream to exert corresponding effects. *CXCL12* may also induce systemic immune responses by interacting with angiotensin-converting enzyme 2, potentially explaining why patients with SCI or TBI often develop NHO alongside systemic metabolic abnormalities (Zheng et al., 2025). *In vitro* cell experiments further identified M1-type macrophages as the core source of *CXCL12* secretion in SCI-induced NHO, challenging previous understanding of the *CXCL12* secretion source. *CXCL12* is primarily secreted by fibroblasts, endothelial cells, and neutrophils, with its functions including promoting cell migration, wound healing, and immune regulation (Li et al., 2023; Sun et al., 2023; Zhou et al., 2025). In contrast, M1 macrophages predominantly secrete pro-inflammatory factors (e.g., IL-1β, IL-6, tumor necrosis factor-α) and chemokines (e.g., *CXCL9*, *CXCL10*), with insufficient evidence supporting their role as the primary source of *CXCL12* secretion (Dai et al., 2020; Wu et al., 2022). Gene expression profile analyses have revealed that M1 macrophages exhibit prominent *CXCL10* overexpression, whereas *CXCL12* secretion remains unsubstantiated (Dai et al., 2020; Wu et al., 2022). This study reveals that following M1 polarization, not only is intracellular *CXCL12* mRNA expression upregulated by approximately 20-fold, but the concentration of *CXCL12* in the secreted supernatant is also significantly increased compared with undifferentiated macrophages. Specific inhibition of *CXCL12* secretion via siRNA reduces the proliferation activity, migration efficiency, and osteogenic capacity of BMSCs induced by M1-type macrophages, suggesting that *CXCL12* is a key effector molecule regulating BMSC behavior in M1-type macrophages (Li et al., 2023; Sun et al., 2023).

The *PI3K/AKT* pathway demonstrated multilevel regulatory effects on BMSCs. In this study, we demonstrated that the PI3K/AKT pathway, as a downstream hub of the *CXCL12*-*CXCR4* signaling pathway, directly mediates the osteogenic differentiation of BMSCs, and activation of this pathway upregulates the expression of RUNX2 and OCN. At the cellular interaction level, activation of the *PI3K/AKT* pathway regulates the migration, proliferation, and osteogenic differentiation of BMSCs (Sun et al., 2020; Li et al., 2022). At the molecular mechanism level, the *PI3K/AKT* pathway promotes late-stage cartilage regeneration in MSCs through the TGF-β–mothers against decapentaplegic homolog 2/SOX9 axis (Klampfleuthner et al., 2022) and synergistically influences osteoblastic differentiation by regulating macrophage polarization (Zhao et al., 2020; Lu et al., 2022). At the microenvironment remodeling level, the *PI3K/AKT* pathway not only directly participates in the fibrotic process (e.g., promoting collagen deposition) (Wang et al., 2022) but also maintains the oxidative stress state of BMSCs by activating autophagy and inhibiting ferroptosis (Zhao et al., 2021; Lan et al., 2022). Additionally, enhanced *PI3K/AKT* phosphorylation levels in SCI mouse models are positively correlated with spasticity, whereas pathway inhibitors can significantly alleviate spasticity (Yang et al., 2019). This finding aligns with Xie et al. (2020), who identified spasms as a risk factor for NHO after SCI, suggesting that targeting and inhibiting this pathway could suppress NHO after SCI. Furthermore, this study showed that *CXCL12* binding to *CXCR4* receptors promotes the migration, proliferation, and osteoblastic differentiation of BMSCs by activating the *PI3K/AKT* pathway. Blocking the relevant pathway weakens the effect of CXCL12. The *CXCL12-CXCR4* signaling axis influences the *PI3K/AKT* pathway, thereby enhancing the migration capacity and proliferative activity of BMSCs (Liu et al., 2022; Zhang et al., 2022). Whether the *CXCL12-CXCR4* signaling axis induces osteogenic differentiation of BMSCs by promoting *PI3K/AKT* pathway activation has not been reported.

This study has the following limitations that require clarification: First, regarding the animal model, although an NHO model was established following SCI, the specific mechanisms linking spasticity to ectopic ossification have not been thoroughly elucidated. In particular, quantitative detection of key neurotransmitters, such as substance P, osmotin, and calcitonin gene-related peptide, as well as nerve growth factor, has not been conducted. This lack of quantitative data leaves insufficient experimental evidence for a direct regulatory role of neural factors in the ossification process. Second, the evaluation system is limited. Existing behavioral tests and ROM measurement methods are insufficient to comprehensively assess key clinical indicators such as neuropathic pain, sensory function grading, and bladder function, which partially compromises the model’s integrity. Finally, experimental validation remains incomplete. Current findings regarding the *CXCL12-CXCR4-PI3K/AKT* signaling axis primarily rely on *in vitro* results, and *in vivo* mechanisms require further validation through more systematic animal studies. These limitations point to areas for improvement in future research.

**Figure 8:**
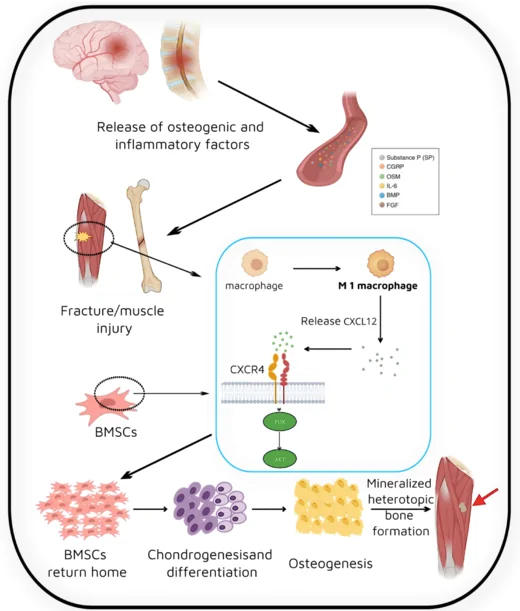
M1 macrophages promote osteogenic differentiation of BMSCs through the *CXCL12-CXCR4-PI3K-AKT* signaling axis, mediating heterotopic ossification following spinal cord injury. Created with BioRender.com. AKT: AKT serine/threonine kinase; BMP: bone morphogenetic protein; BMSCs: bone marrow mesenchymal stem cells; CGRP: calcitonin gene-related peptide; CXCL12: C–X–C motif chemokine ligand 12; CXCR4: C–X–C motif chemokine receptor 4; FGF: fibroblast growth factor; IL-6: interleukin-6; OSM: oncostatin M; PI3K: phosphatidylinositol 3-kinase; SP: substance P.

In conclusion, this study established a complete pathological mechanism chain of NHO following SCI. SCI induces a neuroinflammatory state, and when muscle tissue is injured, it promotes the aggregation of macrophages at the injury site, which then polarize into the M1 type and secrete *CXCL12*. *CXCL12* binds to the CXCR4 receptor on the surface of BMSCs, activating the *PI3K/AKT* pathway and enhancing BMSC proliferation, migration, and osteoblastic differentiation capabilities, ultimately leading to NHO after SCI. The cascading amplification effect of this signaling pathway spans the entire process of NHO formation after SCI, exerting significant regulatory effects from the shaping of the inflammatory microenvironment to the bone matrix mineralization stage. This study provides theoretical support for elucidating the pathogenesis of NHO following SCI and developing therapeutic strategies on the basis of macrophage reprogramming or *CXCL12/PI3K/AKT* inhibitors (mechanism illustrated in **Figure 8**).

## Additional file:

***Additional Figure 1:*** *M1 polarization induces macrophages.*

Additional Figure 1 M1 polarization induces macrophages.
(A) Flow cytometric scatter plots of the M0 and M1 groups. (B) Relative proportions of CD86-positive cells. Data are expressed as mean ± standard error (*n* = 9/group). ^***^P < 0.001 (unpaired two-tailed *t*-test). AKT: AKT serine/threonine kinase; BMP: bone morphogenetic protein; BMSCs: bone marrow-derived mesenchymal stem cells; CGRP: calcitonin gene-related peptide; CXCL12: C-X-C motif chemokine ligand 12; CXCR4: C-X-C motif chemokine receptor 4; FGF: fibroblast growth factor; IL-6: interleukin-6; NC: normal control; OSM: oncostatin M; PI3K: phosphatidylinositol 3-kinase.

## Data Availability

*All data generated or analyzed in this study are included in this published article and its Additional file.*
